# Supplementary figures and images for: Ancient DNA analysis of archaeological specimens extends Chinook salmon’s known historic range to San Francisco Bay’s tributaries and southernmost watershed
Source: PLoS One. 2021 Apr 15;16(4):e0244470. doi: 10.1371/journal.pone.0244470 (PMC8049268; doi:10.1371/journal.pone.0244470)

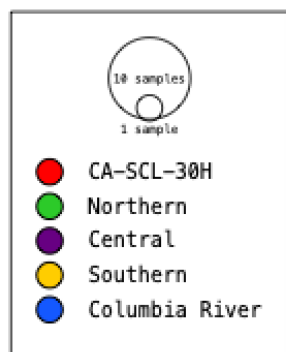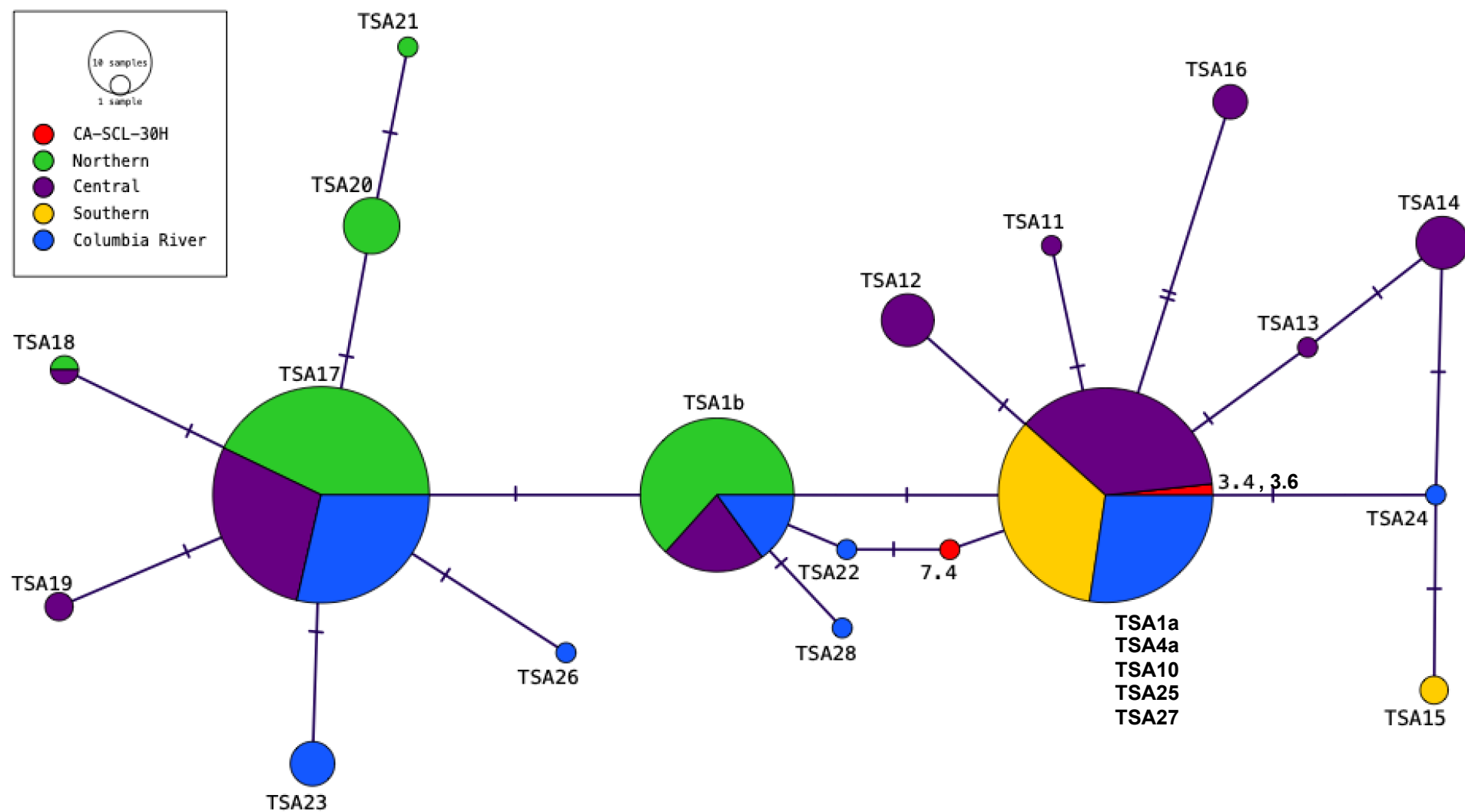

Supplement: S1 Fig — (PDF) [file pone.0244470.s001.pdf]
