# Supplementary material for: Ancient DNA analysis of archaeological specimens extends Chinook salmon’s known historic range to San Francisco Bay’s tributaries and southernmost watershed
Source: PLoS One. 2021 Apr 15;16(4):e0244470. doi: 10.1371/journal.pone.0244470 (PMC8049268; doi:10.1371/journal.pone.0244470)
Supplement: S1 File — (DOCX) [file pone.0244470.s002.docx]

Supporting Information for

**Ancient DNA Analysis of Archaeological Specimens Extends Chinook Salmon’s Known Historic Range to San Francisco Bay’s Tributaries and Its Southernmost Watershed**

Richard B. Lanman^1,2^*, Linda Hylkema^3^, Cristie M. Boone^4^, Brian Allée^5^, Roger O. Castillo^6^, Stephanie A. Moreno^1^, Mary Faith Flores^7^, Upuli DeSilva^7^, Brittany Bingham^7^, Brian M. Kemp^7^

*Correspondence to: [ricklanman@gmail.com](mailto:ricklanman@gmail.com) (R.B.L.)

**Supporting Information**

**Description of Excavation Site**

CA-SCL-30H is located in Santa Clara, Santa Clara County, California, within the SE ¼ of the NW ¼ of the NE ¼ of Section 2, T. 7S. R. 1W on the San Jose West 7.5’ Quadrangle (PR 1980) [1]. The present drainage of the Guadalupe River is 2.5 km (1.5 miles) due east of the site and Saratoga Creek drainage lies approximately 2 miles to the west. The Third Mission Santa Clara is located approximately 3 miles to the northwest of downtown San Jose (Old Pueblo de San Jose). The elevation of the site is approximately 70 feet above mean sea level.

**Legal Entitlement Process for Santa Clara University Archaeological Work at Mission Santa Clara**

Santa Clara University (SCU) published a Final Environmental Impact Report (FEIR) for a Ten Year Capital Plan in early 2003. The Ten Year Capital Plan outlined a proposed building plan that called for several campus improvement projects, including improvements or additions for numerous locations across campus. This included development within the areas defined archaeologically as the Mission Santa Clara Rancheria and that are the subject of this article.

The FEIR provided information concerning the environmental consequences of SCU’s building plan (SCU 2003). A Draft EIR was sent for comment to several local and state-wide governing agencies. Comments were received from: the California Department of Transportation (Caltrans); the California Regional Water Quality Control Board; the Santa Clara Valley Transportation Authority; the City of Santa Clara Historical and Landmarks Commission; the City of Santa Clara Planning Commission Cultural Resources Sub-Committee; and Santa Clara University. The FEIR conformed to guidelines set forth by the California Environmental Quality Act (CEQA). It stated that if the proposed capital plan had a significant effect on the environment, specific actions would be taken to mitigate or avoid those impacts. The City of Santa Clara is a Certified Local Government. As such, it has review authority for all cultural documents written on sites within City boundaries, including those on the SCU campus. A Cultural Resources Treatment Plan was drafted in part based on previous plans and research designs written by the various team members, and was also derived from other urban area Treatment Plans written in the last decade in the San Francisco Bay region (references available upon request).

**Ancient DNA Sequencing Methods**

All pre-polymerase chain reaction (PCR) activities were conducted in the ancient DNA (aDNA) facility at the Laboratories of Molecular Anthropology and Microbiome Research (LMAMR) at the University of Oklahoma. This facility is a dedicated workspace for processing aged, degraded, and/or low copy number DNA samples. Precautions aimed to minimize and monitor the introduction of contamination are practiced in the laboratory.

**DNA Extraction**

DNA was extracted from 88 salmonid vertebrae. Eighty-one vertebrae were small (~2-4 mm in diameter), relatively complete, and weighed 0.3-25.2 mg. The whole of each of these samples was exhausted for DNA extraction. Five vertebrae were larger, but largely incomplete. Sample 3.4 weighed 49.2 mg, sample 3.6 weighed 27.9 mg, and sample 7.4 weighed 5.9 mg, and these were exhausted for DNA extraction. From the remaining two samples (1.5 and 3.3) we carefully subsampled each from the whole 56 mg and 50.4 mg of bone, respectively, with a single use razor blade. All samples were submerged in 6% (w/v) sodium hypochlorite for 4 minutes [2]. The sodium hypochlorite was poured off and the samples were quickly submerged in DNA-free water twice.

The bone samples were transferred to 1.5 mL tubes, to which aliquots of 500 µl of 0.5M EDTA were added, and the tubes gently rocked at room temperature for >48 hours. An extraction negative control, to which no bone material was added, accompanied each batch of extractions.

DNA was extracted following the method previously described [3]. Ninety µl of proteinase K (BIOBASIC cat # 32181) at a concentration of 1 mg/30 µl (or >20 Units/30 µl) were added to each sample, and the tubes incubated at 64-65^º^C for 3 hours. Following proteinase K digestion, the tubes were centrifuged at 15,000 revolutions per minute (rpm) for one minute to pellet any undigested bone, dirt, and/or “sludge”. The volumes of liquid were moved to new 1.5 mL tubes, to which 750 µl of 2.5% “resin” (i.e., 2.5% celite in 6 M guanidine HCl) and 250 µl of 6 M guanidine HCl were added. The tubes were vortexed multiple times over approximately a 2 min period.

Promega Wizard minicolumns were attached to 3 mL luer-lok syringe barrels (minus the plunger) and placed on a vacuum manifold. Three mL of DNA-free water were first pulled across the columns with the intent to wash away potential contaminating DNA. The DNA/resin mixture was subsequently pulled across the columns. The silica pelleted on the minicolumns was then rinsed by pulling 3 mL of 80% isopropanol across the columns.

The minicolumns were then placed in new 1.5 mL tubes and centrifuged at 10,000 rpm for 2 minutes to remove excess isopropanol. The minicolumns were transferred to new 1.5 mL tubes. Fifty µl of DNA-free water heated to 64-65^º^C were added to the minicolumns and left for 3 minutes before centrifugation of the tubes for 30 seconds at 10,000 rpm. This step was repeated, amounting to 100 µl of extracted DNA. Ten microliters of the full concentration eluates and extraction negative controls were diluted 1:10 and used in PCR, as described below.

**Inhibition Test and Repeat Silica Extraction**

The full concentration DNA eluates were tested for the presence of PCR inhibitors following Kemp et al.’s [3] rationale using a “turkey collective” as the aDNA positive control. The control, in this case, was DNA pooled from that extracted from various archaeological turkey (*Meleagris gallopavo*) bones [4]. The choice to pool these individual extractions was made with the intention to reduce variation between turkey DNA eluates in both endogenous mitochondrial DNA copy number and possible inhibitors co-extracted with the turkey DNA. Before they are used in experiments, each turkey collective was demonstrated to PCR amplify consistently (in 6 or more amplifications), hence serving as a positive control.

Fifteen microliter inhibition test PCRs amplified a 186 bp portion of turkey displacement loop (D-loop) using the primers “T15709F” and “T15894R” as previously described [4]. The components of these PCRs were as follows: 1X Omni Klentaq Reaction Buffer (including a final concentration of 3.5 mM MgCl_2_), 0.32 mM dNTPs, 0.24 µM of each primer, 0.3 U of Omni Klentaq LA polymerase, and 1.5 µl of turkey collective DNA. These reactions were spiked with 1.5 µl of potentially inhibited, full concentration DNA eluates from the salmonid vertebrae. The extraction negative controls were also tested for inhibitors in this manner. These PCRs were run in parallel with reactions that contained only turkey collective DNA (i.e., were not spiked). These reactions served as positive controls and allowed us to preclude PCR failure from contributing to our results. PCR negatives also accompanied each round of amplification, allowing us to monitor for possible contamination. Following denaturing at 94^º^C for 3 minutes, 60 cycles of PCR was conducted at 94^º^C for 15 s, 60^º^C for 15 s, and 68^º^C (note that this is the optimal extension temperature for Omni Klentaq LA polymerase) for 15 s. Finally, a 3-minute extension period at 68^º^C was conducted prior to bringing the reactions to 10^º^C.

If the turkey collective failed to amplify when spiked with any given ancient salmonid DNA eluate, we considered the eluate to be inhibited. In the case that spiking the ancient DNA permitted amplification of the turkey collective DNA, we considered that DNA eluate to be inhibitor “free”.

Full concentration eluates deemed to be inhibited in the manner just described were subjected to repeat silica extraction [3]. To the remaining volume of the eluate, 750 µl of 2.5% resin and 250 µl of 6 M guanidine HCl were added. The samples were vortexed numerous times over a 2 min period. The extraction then followed procedures described above, except that the volume used to elute the DNA from column matched the volume being repeat silica extracted. For example, if the starting volume was 87 µl, 43.5 µl of DNA-free water heated to 65^º^C was added to the minicolumns and left for 3 minutes before centrifugation. This step was repeated twice for a total volume of 87 µl.

These repeat silica eluates were tested again for inhibition as described above. Those still deemed to be inhibited were once again repeat silica extracted and tested again for inhibition. This was carried out until all full concentration eluates were deemed to be uninhibited.

**Fish Species Identification and PCR**

For fish species identification we used primers previously described by Jordan et al. [5] that amplify an 189 bp section of the mitochondrial 12S gene [relative to a rainbow trout (*Oncorhynchus mykiss*), reference sequence (Genbank accession number DQ288271.1) [6]. The 148 bp sequences produced from these amplicons are particularly useful for discriminating between salmonid species, but have also been demonstrated to be useful for identifying a wide range of non-salmonid species [7]. Note that Jordan et al. originally described their reverse primer in the wrong orientation [5]. The corrected primers are OST12S-F (5’-GCTTAAAACCCAAAGGACTTG-3’) and OST12S-R (5’-CTACACCTCGACCTGACGTT-3’).

Full concentration eluates (deemed to be uninhibited) and the 1:10 dilutions of the original full concentration DNA eluates were subjected to PCR amplification in 15 µl reactions as follows. First, “standard” PCRs contained 1X Omni Klentaq Reaction Buffer, 0.32 mM dNTPs, 0.24 µM of each primer, 0.3 U of Omni Klentaq LA polymerase, and 1.5 µl of template DNA.

Second, we employed PCR enhancer cocktail-P (PEC-P; DNA Polymerase Technology) that has been found useful in amplifying aged and degraded DNA [8]. Fifteen µl PEC-P PCR reactions with Klentaq contained 1X Omni Klentaq Reaction Buffer, 0.32 mM dNTPs, 0.24 µM of each primer, 0.3 U of Omni Klentaq LA polymerase, 20% (v/v) PCR enhancer cocktail, and 1.5 µl of template DNA. Fifteen µl PEC-P PCR reactions with Platinum Taq contained 1X PCR Buffer, 1.5 mM MgCl_2_, 0.32 mM dNTPs, 0.24 µM of each primer, 0.3 U of Platinum Taq, 20% (v/v) PCR enhancer cocktail, and 1.5 µl of template DNA.

Third, rescue PCR at a 25% increase was carried out as described by Johnson and Kemp [9]. Rescue PCRs contained 1.25X Omni Klentaq Reaction Buffer (including a final concentration of 4.375 mM MgCl_2_), 0.4 mM dNTPs, 0.3 µM of each primer, 0.375 U of Omni Klentaq LA polymerase, and 1.5 µl of template DNA.

When employing Klentaq, PCR conditions were as follows: denaturing at 94^º^C for 3 minutes followed by 60 cycles of PCR conducted at 94^º^C for 15 s, 55^º^C for 15 s, and 68^º^C for 15 s. Finally, a 3 minute extension period at 68^º^C was conducted prior to bringing the reactions to 10^º^C. When employing Platinum Taq, extension temperatures were 72^º^C.

Positive amplification was confirmed by separating 2 µl of amplicons on 2% agarose gels stained with Gelred and visualized with UV light. Amplicons were sequenced in both directions at Genewiz ([www.genewiz.com](http://www.genewiz.com)). Sequences were aligned to a rainbow trout (*O. mykiss*) full mitochondrial genome reference sequence (Genbank accession DQ288271.1) [6] in Sequencher v5.4.6. Salmonid species determinations were made following Jordan et al. (see their Table 2) [5].

**Additional Chinook salmon (*O. tshawytscha*) PCR Amplification and Sequencing**

Based on the 12S species identification procedures described above, three specimens (numbers 3.4, 3.6, and 7.4) were identified as Chinook salmon (*O. tshawytscha*). In order to gain additional phylogeographic information about these specimens, we employed the primers previously described to sequence a portion of the mitochondrial genome encompassing a 563 bp stretch from the end of the D-loop through tRNA-Phe and into the 12S gene [10].

Four forms of PCR, as described above, were used to produce amplicons with these primer sets: 1) standard PCR, 2) PEC-P PCR with Klentaq, 3) PEC-P PCR with Platinum Taq, and 4) Rescue PCR. Here we also used 1.5 or 3.0 µl of template DNA in the reaction with Klentaq. Sixty cycle PCRs were conducted as described above using the annealing temperatures described previously [10].

Sequences were aligned to a Chinook full mitochondrial genome reference sequence (Genbank accession number NC_002980.1) [6] in Sequencher v5.4.6. Salmonid species determinations were made following Jordan et al. (see their Table 2) [5].[11]

**Median-joining Network Construction**

The sequences of the D-loop spanning into the 12S gene from specimens 3.4, 3.6, and 7.4 (Genbank accession numbers MW113717–MW113719) were aligned with comparative Chinook sequences from Martin et al. and Johnson et al [10,12]. The combined data set was truncated to nucleotide positions (nps) 573-1121, relative to a comparative full mitochondrial genome of Chinook salmon (Genbank accession number NC_002980.1). Haplotype “TSA2a” described by Martin et al. [12] was removed from this analysis due to missing data (Ns) at nps 587 and 612. DNA Alignment (version 1.3.3.2) was used to convert the data to a Nexus file, which was then imported into PopART (version 1.724; [13]). A median-joining network was created using the default parameters in PopART [11]. Insertions and deletions were omitted from network construction.

**Results of aDNA Amplification and Sequencing of the Three Chinook Salmon Samples and Median-Joining Network Construction**

Specimen 3.4 exhibits the following mutations: 592delG, T596A, 608delT, 884delG, 974.1C, and T1028G. A BLAST search of this haplotype at NCBI reveals a 100% match to a Chinook salmon haplotype called “TSA 1A”, one that has previously been observed in Chinook salmon originating from rivers located in the states of Washington, Oregon, and California (United States of America) [12]. Specimen 3.6 exhibits the following mutations: 592delG, T596A, 608delT, 884delG, T1028G. A BLAST search of this haplotype in reveals a 100% match to a Chinook salmon haplotype called “TSA 10” that has previously been observed in Chinook salmon originating from rivers in the same states [12]. Specimen 7.4 exhibits the following mutations: 592delG, T596A, 608delT, G869A, 884delG, 974.1C, T1028G. A BLAST search of this haplotype reveals it to be unique. However, it is one mutational step from “TSA 1” (which lacks the lacks G869A mutation) and one mutational step from “TSA 22 (which is derived by a G641A mutation). These are the two closest related lineages in Genbank. “TSA 22” has been observed in prehistoric Chinook salmon from the Columbia River [10].

The median-joining network is found in S1 Fig. This analysis further supports close mitochondrial relations between the Chinook sampled from Mission Santa Clara de Asís (CA-SCL-30H) and other Chinook lineages observed throughout their range. This lends further credibility to our results, as they make phylogenetic sense [14].

**S1 Fig. Median-joining network.**

**References for Supporting Information**

1. Peelo, S., Hylkema, L., Blount, C., Garlinghouse, T., Ellison, J., Boone, C. M., et al. The Indian Rancheria at Mission Santa Clara de Asis: Cultural Resources Mitigation for the Edward M. Dowd Art and Art History Building and Parking Structure, edited by S. Peelo and L. Hylkema. Santa Clara University Cultural Resource Management Heritage Stewardship Reports, L. Hylkema, general editor. Santa Clara, California: Santa Clara University; In Press.

2. Barta JL, Monroe C, Kemp BM. Further evaluation of the efficacy of contamination removal from bone surfaces. Forensic Sci Int. 2013;231: 340–348. doi:10.1016/j.forsciint.2013.06.004

3. Kemp BM, Monroe C, Judd KG, Reams E, Grier C. Evaluation of methods that subdue the effects of polymerase chain reaction inhibitors in the study of ancient and degraded DNA. J Archaeol Sci. 2014;42: 373–380. doi:10.1016/j.jas.2013.11.023

4. Kemp BM, Judd K, Monroe C, Eerkens JW, Hilldorfer L, Cordray C, et al. Prehistoric mitochondrial DNA of domesticate animals supports a 13th century exodus from the northern US southwest. Caramelli D, editor. PLOS ONE. 2017;12: e0178882. doi:10.1371/journal.pone.0178882

5. Jordan LG, Steele CA, Thorgaard GH. Universal mtDNA primers for species identification of degraded bony fish samples: Molecular Diagnostics and DNA Taxonomy. Mol Ecol Resour. 2010;10: 225–228. doi:10.1111/j.1755-0998.2009.02739.x

6. Brown KH, Drew RE, Weber LA, Thorgaard GH. Intraspecific variation in the rainbow trout mitochondrial DNA genome. Comp Biochem Physiol Part D Genomics Proteomics. 2006;1: 219–226. doi:10.1016/j.cbd.2005.11.004

7. Grier C, Flanigan K, Winters M, Jordan LG, Lukowski S, Kemp BM. Using ancient DNA identification and osteometric measures of archaeological Pacific salmon vertebrae for reconstructing salmon fisheries and site seasonality at Dionisio Point, British Columbia. J Archaeol Sci. 2013;40: 544–555. doi:10.1016/j.jas.2012.07.013

8. Kemp BM, Bingham B, Frome R, Labonte M, Palmer E, Parsons ES, et al. Subduing the influence of PCR inhibitors on amplifying aged, degraded, and low copy number DNA: PCR enhancer cocktail-p and rescue PCR. Kalendar R, editor. PLOS ONE. 2020;15: e0234745. doi:10.1371/journal.pone.0234745

9. Johnson BM, Kemp BM. Rescue PCR: Reagent-rich PCR recipe improves amplification of degraded DNA extracts. J Archaeol Sci Rep. 2017;11: 683–694. doi:10.1016/j.jasrep.2017.01.006

10. Johnson BM, Kemp BM, Thorgaard GH. Increased mitochondrial DNA diversity in ancient Columbia River basin Chinook salmon Oncorhynchus tshawytscha. Chiang T-Y, editor. PLOS ONE. 2018;13: e0190059. doi:10.1371/journal.pone.0190059

11. Bandelt HJ, Forster P, Rohl A. Median-joining networks for inferring intraspecific phylogenies. Mol Biol Evol. 1999;16: 37–48. doi:10.1093/oxfordjournals.molbev.a026036

12. Martin KE, Steele CA, Brunelli JP, Thorgaard GH. Mitochondrial Variation and Biogeographic History of Chinook Salmon. Trans Am Fish Soc. 2010;139: 792–802. doi:10.1577/T09-080.1

13. Leigh JW, Bryant D. popart : full‐feature software for haplotype network construction. Nakagawa S, editor. Methods Ecol Evol. 2015;6: 1110–1116. doi:10.1111/2041-210X.12410

14. Gilbert MTP, Bandelt H-J, Hofreiter M, Barnes I. Assessing ancient DNA studies. Trends Ecol Evol. 2005;20: 541–544. doi:10.1016/j.tree.2005.07.005
